# Military Tract Association

**Published:** 1867-01

**Authors:** A. H. Thompson, Geo. H. Scott

**Affiliations:** Pres’t.; Sec’y.


### MILITARY TRACT ASSOCIATION.

---

The Association met in semi-annual session, at Galesburg, on Tuesday, 11th day of December, 1866, at the hall of the Christian Association, A. H. Thompson, M.D., President, in the chair.

The proceedings of the previous meeting were read and adopted, when the following physicians were presented for membership, and were elected:—Drs. Spalding, Hamilton, Phillips, Woodward, Burlingham, and H. M. Hurd, of Galesburg; Drs. Ewing, Webster, and Crawford, of Monmouth.

It was voted that Dr. McClanahan, of Mercer County, be elected an honorary member, with all the privileges of the Association.

Reading of essays being next in order, Dr. Crossley, of Princeton, read an article on Army Itch, or "Illinois Scratches," which elicited quite an animated discussion.

Dr. Woodward gave a history of the disease, as it prevailed among the soldiers during the late war; and also exhibited some animalculæ recently taken from a patient under his care. He considered the insect essentially different from the true acarus scabiei, but believed the disease amenable to the same treatment. The treatment used by him in the army was, iodide of arsenic internally, and ungt. hyd. oxid. nit. externally.

Dr. Nance believed the disease to be caused by animalculæ, and that the ungt. hyd. fortis, properly applied, was a certain cure.

Dr. Spalding recognized the disease as being identical with scabies; curable with the migt. sulphuris.

Dr. Holton believed the disease to be caused by animalculæ, but differing in the form of eruption produced by the acarus scabiei not infesting the spaces between the fingers and about the joints; both amenable to mercurial treatment; but the difficulty of effecting a cure in the army, was the inability to observe cleanliness, also the frequently depressed physical vigor of the patient.

Moved by Dr. Crossley, and carried, That the disease under consideration is animalcular in origin; and that mercury or sulphur is the treatment.

Dr. Nance, of Kewanee, read an essay on the Endemic and Epidemic Diseases of Henry County, during 1866.

Moved and carried, That the Association return a vote of thanks to Dr. Nance, for his very interesting essay.

Dr. Holton, of Buda, read an interesting paper, entitled "Remarks on Different Subjects," being a detailed history of a variety of cases, of much interest, coming under his care during the past summer.

REPORT OF CASES, AND PRESENTATION OF MORBID SPECIMENS.

Dr. Woodward, of Galesburg, presented the calvaria of a boy, *æt.* 15, coming under his observation while in service in the army, who had been kicked by a horse over the right supra-orbital region four years prior; portions of the frontal bone having been removed by a physician in Madison, Wisconsin, the patient suffering with epilepsy. The peculiar features in the case were, that the convulsions could be produced at any time by pressure upon the cicatrix, and continued as long as the pressure was maintained; and, that during the eighty hours prior to his death, he had two hundred distinct convulsions.

Dr. Nance, of Kewanee, presented a specimen of renal calculus, of the mulberry variety, with a history of the case.

Dr. H. S. Hurd, of Galesburg, reported an exceedingly interesting case of aneurism of the innominate artery, and right sub-clavian.

Dr. Scott, of Kewanee, reported a case of anchylosis and caries of the right elbow-joint, occurring in a boy *æt.* 13, of scrofulous habit; after inflammation of the synovial membrane and cartilages of the joint. When the case came under the Doctor's care, it was of several months' duration. Amputation resulted in recovery. The morbid specimen was exhibited to the Association.

Dr. Holton, Chairman of the Committee of Resolutions, in respect to Prof. Brainard, deceased, reported:—

*Resolved*, That, on account of a dispensation of Providence in removing our brother, Prof. D. Brainard, from our midst, we recognize the hand of Him who is the author of our being, and we desire to say, His will be done.

*Resolved*, That our brother had acquired the reputation of being among the first as a teacher, and as an operator in surgery; and that his character and position for usefulness was world-wide; therefore, we deplore his loss as irreparable.

*Resolved*, That in his life we see encouragement to persevere in our efforts to arrive at a position of distinction, and to improve our ability to alleviate the sufferings of humanity; and we regard him as a worthy example for the profession.

*Resolved*, That we sincerely condole with the remaining members of the family.

[Signed] N. HOLTON, M.D.,
JOHN EWING, M.D., } Committee.
H. S. HURD, M.D.,

Moved, by Dr. Holton, and carried, that a committee of five be appointed, to report on surgery, practice, obstetrics, materia medica, and therapeutics.

The Chair reported the following gentlemen on said committees:—

*Surgery*—Drs. Hamilton and Spalding, of Knox; Crossley, of Bureau; Secord, of Henry; Webster, of Warren.

*Practice*—Drs. Latimer, of Bureau; Hume, of Henry; H. S. Hurd, of Knox; Crawford, of Warren; Boardman, of Stark.

*Obstetrics*—Drs. Nance, of Henry; Holton, of Bureau; Webster, of Warren; Morse and Woodward, of Knox.

*Materia Medica and Therapeutics*—Drs. Ewing, of Warren; Smiley, of Henry; H. M. Hurd, of Knox; Clark, of Henry; Breed, of Bureau.

Moved, by Dr. Nance, and carried, that the President shall notify the committees one month before the meeting of the Association.

The President appointed Drs. Burlingham, of Knox, and Webster, of Warren, on special essays.

Moved, by Dr. Holton, and carried, that the President and Secretary be a committee on publication.

Moved, by Dr. Crossley, and carried, that a vote of thanks be returned to the members of the profession in Galesburg, for their courtesy; also to the Christian Association, for the use of their hall.

Moved, by Dr. Spalding, and carried, that we adjourn, to meet at 10 o'clock A.M., on the second Tuesday in May, 1867, at Monmouth.

A. H. THOMPSON, *Pres't.*

GEO. H. SCOTT, *Sec'y.*

